# MR Imaging‐Based Biomarkers for Strength Prediction: A Statistical Shape and Architecture Modeling of Quadriceps Muscles

**DOI:** 10.1002/jmri.70377

**Published:** 2026-06-11

**Authors:** Salim Bin Ghouth, Ozkan Cigdem, Valentina Mazzoli

**Affiliations:** ^1^ Bernard and Irene Schwartz Center for Biomedical Imaging, Department of Radiology New York University New York New York USA

**Keywords:** knee extension torque, quadriceps muscles, shape and architecture models, shape models

## Abstract

**Background:**

Muscle mass decline, associated with strength decline, is a hallmark of aging. Yet, strength decline greatly exceeds mass decline. This indicates that aspects of muscle quality and architecture—not reflected by mass—also influence force generating capacity. Additionally, shape modeling enables analysis of the shape variations of muscles beyond size.

**Purpose:**

To predict muscle strength using muscle features beyond muscle quantity.

**Study Type:**

Retrospective cross‐sectional study.

**Population:**

Twenty‐four healthy subjects normally distributed over an age range between 30 and 79 years old with a balanced sex distribution (12 female).

**Field Strength/Sequence:**

3 T MRI using multi‐echo Dixon and Stejskal‐Tanner DTI.

**Assessment:**

Shape‐only and shape + architecture models were generated using water‐only and DTI images of the quadriceps. Multiple linear mixed‐effects models were produced using (1) volume, (2) shape‐only, and (3) shape + architecture. Volume was not added to the shape‐only and shape + architecture models. Features reaching statistical significance within the models were retained for further analysis. Models' performance was evaluated using leave‐one‐subject‐out (LOSO) cross‐validation (CV).

**Statistical Tests:**

Pairwise, subject‐level bootstrapping comparison was conducted and ∆*R* ^2^ and ∆RMSE with 95% confidence interval (CI) were calculated. The improvement was considered statistically significant when both ∆*R* ^2^ and ∆RMSE are positive and the 95% CI did not contain zero. Positive ∆*R* ^2^ and ∆RMSE indicate an increase in *R* ^2^ and a decrease in RMSE values.

**Results:**

Shape‐only features demonstrated an improvement in the model performance compared to muscle volume. Models were significantly improved for the vastus lateralis to predict eccentric torque—∆*R* ^2^ = 0.16 (0.01–0.29), ∆RMSE = 5.0 (0.4–9.7); and for the vastus intermedius predicting isometric torque—∆*R* ^2^ = 0.19 (0.02–0.36), ∆RMSE = 6.5 (0.7–12.0). Shape + architecture features did not significantly improve the performance (all *p* ≥ 0.131).

**Data Conclusion:**

Shape‐only models are promising to quantify variations of muscle shape related to force production, and have the potential to develop imaging‐based biomarkers for muscle strength in diseases.

**Evidence Level:**

3.

**Technical Efficacy:**

Stage 2.

## Plain Language Summary

Muscle mass is one of the main determinants of muscle strength. However, the loss of muscle strength in aging populations greatly exceeds the loss of muscle mass. In this study, a new method was used to quantify muscle variations in shape and architecture associated with muscle strength. Prediction of muscle strength using muscle shape features outperformed prediction of muscle strength using muscle volume alone. This accessible approach, which can be automated, can provide an opportunity for the development of early indicators of muscular diseases like sarcopenia.

**Abbreviations**
CI: confidence interval
CSA: cross‐sectional area
CV: cross validation
DTI: diffusion tensor imaging
LOSO: leave‐one‐subject‐out
MRI: magnetic resonance imaging
PCA: principal component analysis
PCSA: physiological cross‐sectional area
RF: rectus femoris
RMSE: root‐mean‐squared error
SSM: statistical shape modeling
VI: vastus intermedius
VL: vastus lateralis
VM: vastus medialis

## 1 Introduction

Age‐related loss of muscle mass is the strongest predictor of strength decline in older adults [1]. However, it does not fully account for the reduction in muscle strength throughout the lifespan, which becomes extremely severe in debilitating syndromes like sarcopenia. Sarcopenia is a key component of frailty and is associated with an increased risk of falls and hospitalization in aging populations leading to a high risk of morbidity and mortality [1, 2, 3, 4, 5]. According to the European Working Group on Sarcopenia in Older People (EWGSOP2) and the Global Leadership Initiative in Sarcopenia (GLIS), sarcopenia is characterized by decreased muscle strength, reduced muscle quantity, and lower muscle quality; and is accompanied by physical impairments at severe sarcopenia [6, 7]. Besides changes in muscle mass and architecture, sarcopenia is also characterized by fatty infiltration—myosteatosis—and deposition of connective tissue—myofibrosis [8, 9]. Additionally, preferential atrophy of the Type 2 fibers is observed in sarcopenia and contributes to the decline in muscle strength in older adults [10]. This suggests that additional factors, including compositional changes, changes in muscle shape, and internal remodeling, likely precede measurable loss of muscle mass and contribute to suboptimal force generation during both “normal aging” and sarcopenia [11, 12].

Aging processes do not only affect global muscle mass, but also lead to an internal remodeling of fibers and fascicles within the muscle, which directly impacts the muscle force generating capacity [13]. Importantly, muscles with the same volume but different shapes can have different PCSAs and therefore different behaviors in terms of peak force production. However, while muscle CSA and volume can be easily measured with several imaging techniques, the determination of PCSA requires architectural parameters such as pennation angle and fiber length, which are less commonly assessed [14]. As aging processes are known to lead to modifications in terms of fiber architecture, including shortening of muscle fibers and decrease in pennation angle [14, 15], the analysis of these features could enhance predictive models of muscle strength derived from imaging data.

Medical imaging modalities including ultrasound and computed tomography (CT) are useful for quantifying properties of skeletal muscles like muscle volume, length, and cross‐sectional area. Magnetic resonance imaging (MRI) offers high‐resolution assessments of muscle volume and composition, and provides insight into muscle fiber architecture using diffusion tensor imaging (DTI) [16]. DTI is an MRI‐based technique that can reconstruct the complex 3D arrangement of muscle fibers by measuring the diffusion of water molecules in anisotropic tissue such as skeletal muscles. These data allow fiber tractography, whereby the leading diffusion eigenvector delineates local fiber orientation, enabling reconstruction of 3D muscle fibers and estimation of parameters like pennation angle and fascicle length. While the utilization of DTI to study skeletal muscles is rapidly growing, the current analysis methods often focus on average fascicle length or pennation angle within a region of interest, and do not allow for a full depiction of structural complexity [13, 14, 15].

Statistical shape modeling (SSM) is an imaging‐based approach to describe variations of complex anatomical structures within a population by a mean shape and a set of modes of variations, principal components [17]. SSM has been applied to investigate normal shape variations within a specific muscle, or to classify two groups of interest [18, 19, 20, 21, 22, 23]. Previous studies have successfully applied SSM to analyze anatomical structures such as bones and muscles [18, 19, 20, 21, 22, 23]. For instance, Bin Ghouth et al. used shape models of the soleus muscle to differentiate between soleus shape features of children and adolescents with cerebral palsy and control counterparts [18]. Sutherland et al. developed shape models of the hamstrings muscle to classify rugby players and sprinters [19]. Similarly, Umehara et al. developed shape models of the quadriceps of young adults, and identified specific shape features that correlate with isometric knee extension torque [20], highlighting the predictive potential of SSM‐derived features for muscle strength. However, it is unclear whether these models could generalize to older adults, or if combining shape features and architectural data could enhance strength prediction beyond muscle volume.

Population‐averaged 3D muscle architecture can be used to study local architectural features. Bolsterlee [24] has developed a framework to analyze group‐averaged muscle architecture features between two groups and demonstrated its utility to differentiate between pre‐ versus post‐eight‐week strength training and most‐ versus less‐affected limb in unilateral cerebral palsy, but the approach does not integrate a shape modeling method with muscle architecture. This leaves a knowledge gap in effectively combining shape modeling and architecture information to predict muscle strength. Additionally, prior studies [13, 14, 15, 20], did not compare the predictive capabilities of (a) volume‐only, (b) shape‐only, and (c) shape + architecture models across a wide age range. Filling this gap could lead to the development of early imaging biomarkers for “opportunistic screening” of muscle conditions like sarcopenia. This study focuses on advancing muscle strength prediction by investigating two complementary approaches to go beyond muscle volume analysis: (1) indirect analysis of muscle architecture through the muscle external shape, and (2) direct analysis of local fiber architecture using DTI. Herein, we hypothesized that models based on shape‐only and shape + architecture features will significantly improve the prediction of knee torque compared to muscle volume alone.

## 2 Methods

### 2.1 Subjects Characteristics

This study is a retrospective secondary analysis of prospectively collected data that was originally gathered for purposes other than the present research question [25]. The study was approved by the Institutional Review Board at Stanford University, and subjects signed informed consent before participation. For the original study, 24 subjects (12 females; aged 30–79 years old) were recruited from the general community in Santa Clara County, California, USA using flyers and social media advertisements. Bilateral MRI images of the thigh were obtained and knee extension torque was measured as described in Mazzoli et al. [25]. Subjects met inclusion criteria were between 30 and 80 years old, able to provide informed consent, and able to undergo an MRI scan and strength measurement. Exclusion criteria included incompatibilities with MRI scanning, pregnancy, body mass index (BMI) greater than 35 kg/m^2^, diabetes, hormonal supplementation, recent use of corticosteroids, musculoskeletal pathologies in lower extremities, and abnormal blood coagulation. Details about inclusion/exclusion criteria can be found in the original publication [25].

### 2.2 MRI Protocol

MRI scans of the thigh were collected at 3 T (Signa Premier, GE Healthcare) using a 16‐ element flexible coil (AirCoil, GE Healthcare) and the 12‐ element built‐in posterior array. Subjects were scanned in a feet‐first supine position with their legs stabilized using sandbags to reduce subject motion. The imaging protocol consisted of 6‐point Dixon and DTI with the following parameters. *Dixon*: multi‐echo gradient‐echo sequence, TR = 13.3 ms, TE_1_ = 1.14 ms, ∆TE = 0.95 ms, echo train length (ETL) = 12, matrix size = 256 × 256 × 42 (2 stacks), spatial resolution = 1.76 × 1.76 × 6 mm^3^, number of signal averages = 1, and flip angel = 5^ο^. *DTI*: Stejskal‐Tanner sequence with a single‐ shot EPI readout, b‐value = 400 s/mm^2^, 15 diffusion encoding directions, TR = 3500 ms, TE = 43 ms, matrix size = 126 × 126 × 42 (2 stacks), spatial resolution = 3.6 × 3.6 × 6 mm^3^, and number of signal averages = 3. The acquisition protocol included two partially overlapping stacks for both Dixon and DTI, in order to ensure a hip‐to‐knee coverage of the thigh. Water only images, reconstructed using the Dixon approach, were used to obtain muscle segmentations that is used in the development of shape models.

### 2.3 Torque Measurements

Knee extension torque was measured using an isokinetic dynamometer (HumacNorm). Three different tests were performed: (1) concentric (constant angular velocity of 120°/s), (2) isometric, and (3) eccentric (constant angular velocity of −60°/s). During data collection and after subjects were seated on the dynamometer, a demonstration session was performed to familiarize subjects with the protocol. Subjects were asked to give their maximum force against the dynamometer lever arm. For each testing condition, the subjects performed 5 repetitions, and the maximum torque value was utilized for further analysis.

### 2.4 Fitting, Aligning and Shape‐Only Models

The quadriceps muscles—vastus lateralis (VL), rectus femoris (RF), vastus medialis (VM), and vastus intermedius (VI)—were manually segmented in both legs using the Dixon water scan by an experienced medical researcher—with 2 years of experience. Segmentation reproducibility was assessed by a semi‐automatic segmentation approach where automatic segmentations were produced using MuscleMap Toolbox [26] and reviewed by an independent researcher (SB). The analysis revealed dice score range between 81.7% and 88.4%, indicating good inter‐rater variability—Table S1. Surface meshes of the four muscles of both legs were generated using ITK‐snap [27]. Meshes were processed using pyMeshLab library in python for removal of non‐manifold parts and smoothing [28]. Based on a fundamental assumption that the human body is externally symmetrical, left‐leg meshes were reflected against the sagittal plane so that all meshes in this study's datasets are right‐leg muscles and reflected left‐leg muscles. This reflection step ensures consistency across the entire datasets. The non‐rigid registration requires using a sample muscle mesh as a reference to initiate the registration process. The initial reference muscle mesh was selected qualitatively as a representative mesh based on overall size and mesh quality, in order to minimize element deformations when morphing the reference mesh to varying muscle sizes. This mesh was used as a reference for initial non‐rigid registration using radial basis function implemented in Gias3 [23]. This step is important to initiate the registration process and generate an average muscle mesh of the population. Then, meshes were rigidly aligned with the selected reference mesh. An average muscle mesh was generated for the entire population for each individual muscle and the generated mean mesh was used as a reference for a new iteration of a non‐rigid registration process for optimization. The iterative use of mean mesh as a reference mitigated any preferential bias toward the initially selected mesh reference [29]. RMSE of vertex‐to‐vertex Euclidean distance was calculated to evaluate the fitting errors of the non‐rigid registration. No volume scaling was applied to the muscle meshes in this study. Principal component analysis (PCA) was performed to develop a shape‐only model for each muscle. Every muscle shape‐only model contained a mean muscle and a set of independent shape features, principal components, ordered in a descending order.

### 2.5 Tractography (Fiber Orientations)

3D reconstruction of internal muscle architecture was obtained using DTI data. Processing of DTI images included denoising, Gibbs rings removal, motion correction and registration to the Dixon water images using Dipy python library [30]. DTI images were non‐rigidly registered to water‐only images to correct for EPI‐related and eddy currents distortions. A deterministic tractography algorithm implemented in MRTrix3 [31] was used to generate muscle fiber tracts for each individual muscle with the following parameters: minimum length = 15 mm, maximum length = 150 mm, threshold angle = 45^ο^, step size = 0.5 mm, maximum number of streamlines = 10,000. Fiber tracking was terminated when the maximum number of seeding attempts (1000 × maximum number of streamlines) was reached. Tractography streamlines were constrained by muscle mask in which streamlines were terminated when they reached the boundary of a mask. Muscle tracts were digitized and converted into a vector field representing muscle fiber orientations. Anti‐parallel vectors were flipped and aligned with the dominant vector field. Vectors were calculated based on the coordinates of each two points along each muscle fiber tract and assigned to a midpoint between the two points.

### 2.6 Shape + Architecture Models

**FIGURE 1:**
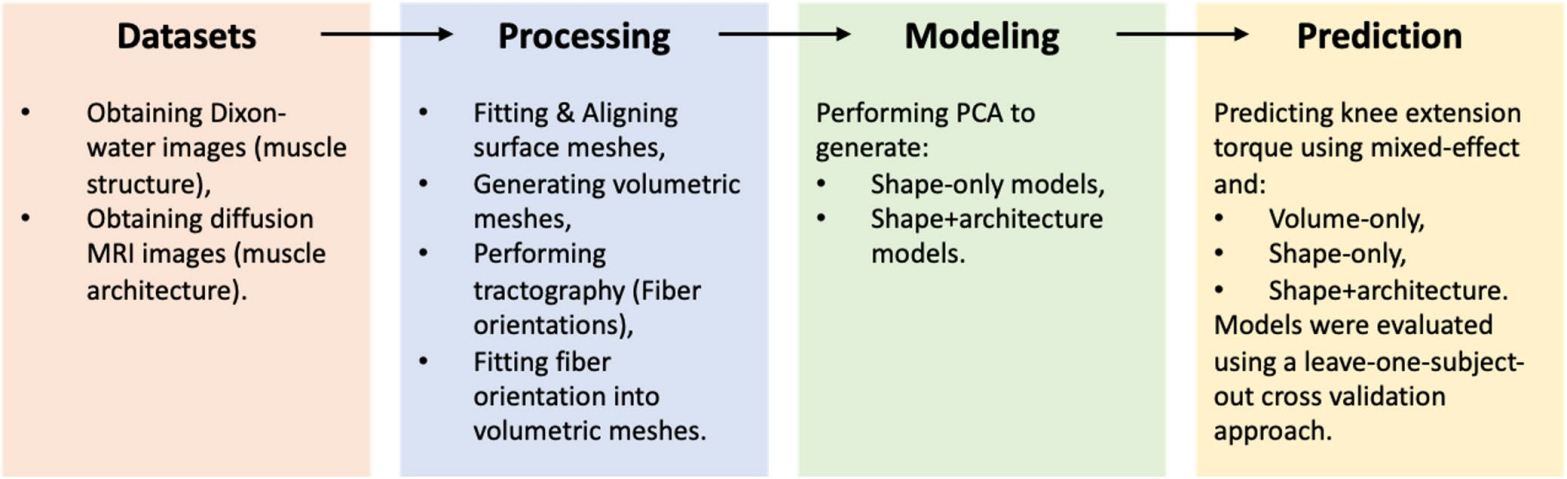
A representation of the workflow used to develop shape‐only and shape + architecture models. The workflow involves fitting and aligning surface meshes, applying principal component analysis (PCA), and using captured shape‐only muscle features to predict muscle strength. The shape + architecture models involved fitting muscle fiber orientations into the aligned volumetric meshes and applying PCA. The regression models, then, were used to predict muscle strength via shape + architecture features.

Registered surface meshes of each muscle obtained from the registration step in the shape‐only models were used to generate registered volumetric meshes. The host‐mesh fitting approach developed by Fernandez et al. [32] was used to complete this step. The host‐mesh fitting process morphed a reference volumetric mesh into every surface mesh in the dataset to generate registered volumetric meshes. Muscle fiber orientations were fitted into their corresponding volumetric mesh using the computed transformation matrices obtained from the registration process. A nearest‐neighbor algorithm was used to determine fiber orientations within each volumetric mesh element. Fiber orientations within each volumetric mesh element were averaged and assigned to the centroid of the element. Six‐by‐N matrices were computed representing the centroid of volumetric mesh elements and their fiber orientations—x, y, z, u, v, w. PCA was used to develop a shape + architecture model represented by a mean muscle model and a small set of independent modes of variations describing the 3D shape and architecture. Figure 1 demonstrates the steps for the development of shape‐only and shape + architecture models.

### 2.7 Torque Prediction

Relationships between muscle strength and PCs obtained from shape‐only and shape + architecture models were investigated using a multiple linear mixed‐effects regression model [33]. PCs were treated as fixed effects to estimate the population‐level association with torque, and subjects were treated as random effects. The dependence of bilateral measurements (right vs. left) per subject was treated as repeated measurements. In particular, the correlations were investigated between (1) concentric (velocity = 120^ο^/s), (2) isometric (velocity = 0), and (3) eccentric (velocity = −60^ο^/s) knee extension torque with features extracted using (1) shape‐only models and (2) shape + architecture models. To establish a comparison against a reference, we also investigated the correlation between torque values and muscle volume. Initially, 10 PCs were considered in the regression models because 10 PCs accounted for more than 94% of the total variance in all shape‐only models and shape + architecture models. Then, PCs were excluded from the regression models based on their corresponding *p* values (*p* ≥ 0.05) as greater *p* values indicate that the relationship between PCs and knee torque, isokinetic and isometric, was likely due to chance.

To evaluate the prediction performance of the models, leave‐one‐subject‐out (LOSO), cross‐validation (CV) was used where one subject was excluded at each iteration and muscle strength for both limbs was predicted. Coefficients of determinations (*R* ^2^) and Root‐mean‐squared error (RSME) with 95% confidence interval (CI) were reported. The correlation between shape features beyond size (PC2 and above) and torque was additionally evaluated after adjusting for volume. In addition, shape‐only and shape + architecture models were adjusted for age, sex, and BMI; and contribution of predictors and subjects' characteristics were analyzed for statistical significance within the models (*p* < 0.05). Bland–Altman analysis was used to evaluate the agreement between the predicted and actual torque values. Our study adheres to the TRIPOD reporting standards, including presentation of data, validation using leave‐one‐out (LOO) CV, performance metrics with 95% CI, as well as the inclusion of limitations and clinical applicability [34].

### 2.8 Statistical Analysis

Pairwise, subject‐level bootstrapping comparisons with 2000 resamples of predicted and actual torque values were conducted, where metrics of ∆*R* ^2^ and ∆RMSE with 95% CI were calculated. The improvement was considered statistically significant when both ∆*R* ^2^ and ∆RMSE were positive and the 95% CI did not contain zero. Positive ∆*R* ^2^ and ∆RMSE indicate an increase in *R* ^2^ and a decrease in RMSE values.

## 3 Results

### 3.1 Shape‐Only Models

Shape‐only models of the four quadriceps muscles—VL, RF, VM, and VI—were developed with an average fitting error using (RMSE) of Euclidian distance of less than 1.5 mm. The shape‐only models described variations of muscle shape using 10 PCs with cumulative variance greater than 94%. This indicates the compactness of the developed models where 10 features can describe more than 94% of the variance of muscle structure. Particularly, the cumulative variance of the first 10 PCs was 94.9% for VL, 97.6% for RF, 97.2% for VM, and 94.6% for VI.

The first PC of the four shape‐only models of the quadriceps muscles explained the largest variance of shape variations (74.1% for VL, 81.2% for RF, 64.8% for VM, and 74.5% for VI). This PC primarily represented variations of muscle size within the study population. This was evidenced by a strong correlation between the first PC in all shape‐only models and muscle volume (*R* ^2^ = 0.79 for VL, 85 for RF, 0.79 for VM and 0.84 for VI).

The second PC of the shape‐only models corresponded to a relationship between length, width, and thickness of each muscle. Along the second PC, muscle variability ranged between thinner‐longer muscle at one end and thicker‐shorter muscles at the other end. Plus, RF muscle presented curvature‐related changes along the second PC. The second PC represented less than 10% of the variations (6.4% for VL, 5.2% for RF, and 5.3% for VI). However, this PC described 21.0% of muscle variability in VM. This indicates that this shape feature, combination of length‐width‐thickness variation, of the VM muscle is more dominant compared to other quadriceps muscles as evidenced by a greater variance.

**FIGURE 2:**
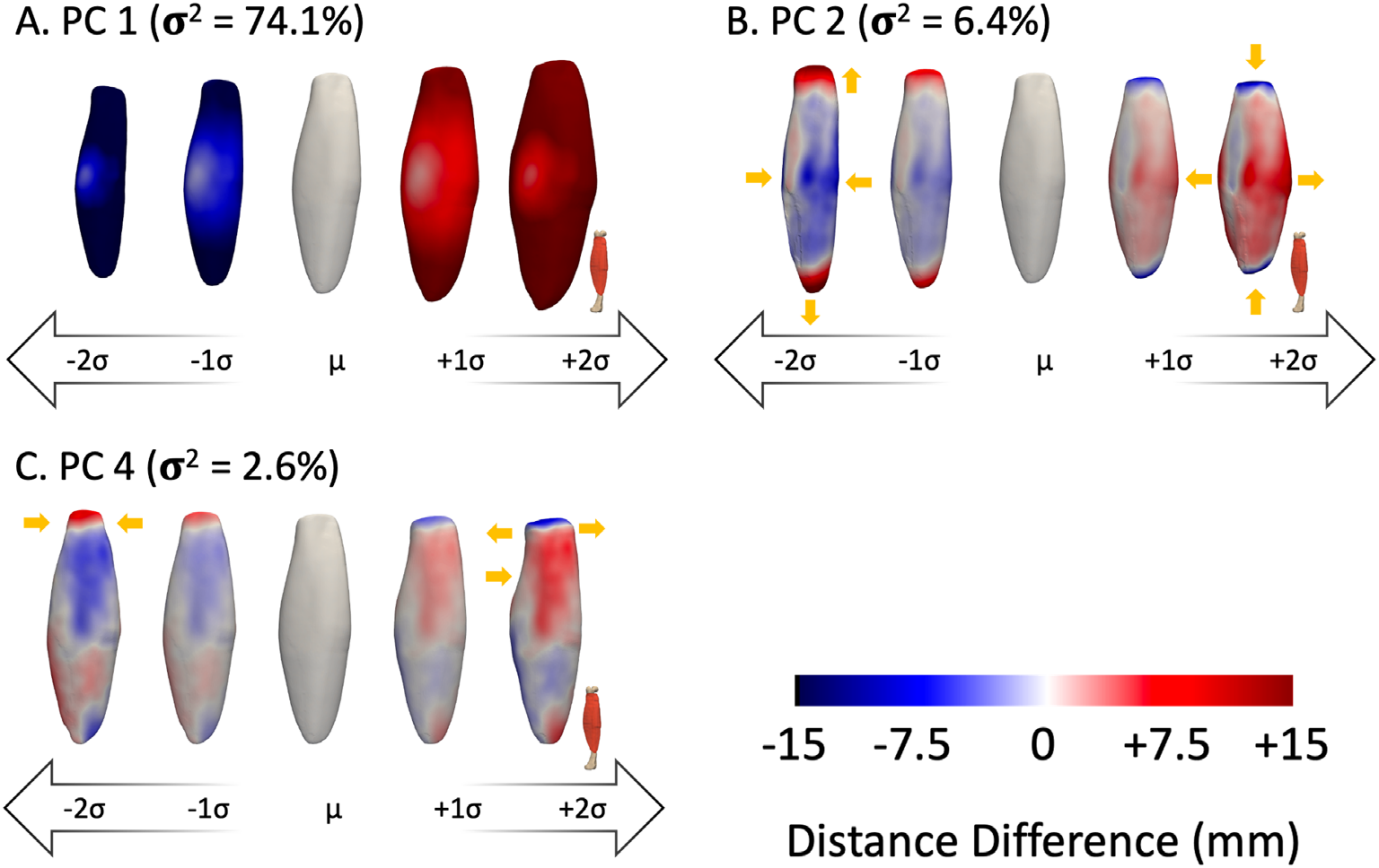
Three principal components (PCs 1, 2, and 4) of the VL muscle contributed to the prediction of concentric knee extension torque (significant shape‐only features, *p* < 0.05). The color‐coded maps represent surface‐to‐surface distance (in millimeters) between the muscle models at ±2 standard deviations and mean muscle. 𝛔^2^ represents the variance of each PC within the shape‐only model. Muscles in all panels are depicted in an anatomical anterior view, as shown in the orientation reference at the bottom right corner of each PC illustrations.

**FIGURE 3:**
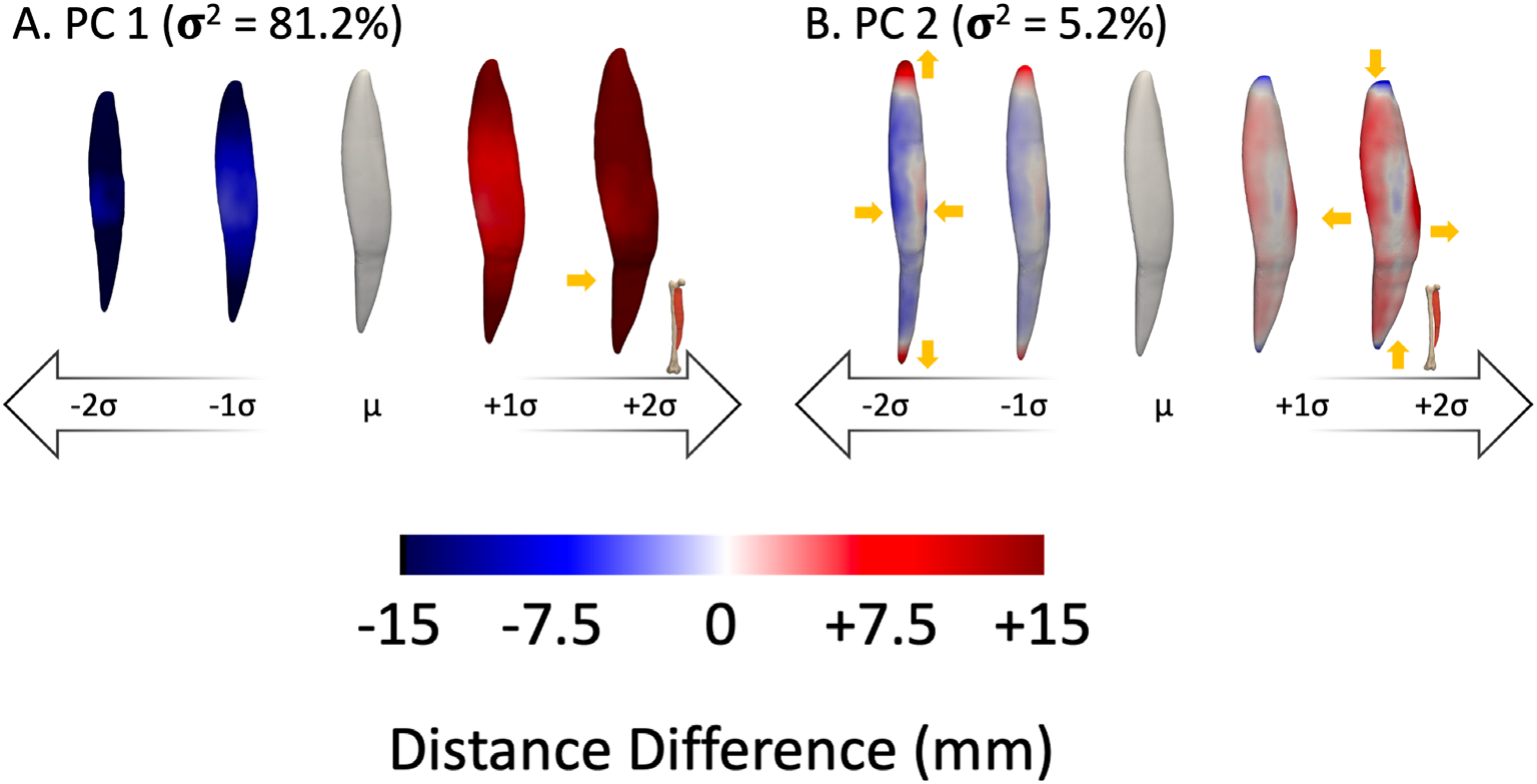
The figure shows two principal components (PCs 1 and 2) of the RF muslce contributed to the prediction of concentric knee extension torque (significant shape‐only features, *p* < 0.05). The color‐coded maps represent surface‐to‐surface distance (in millimeters) between the muscle models at ±2 standard deviations and mean muscle. 𝛔^2^ represents the variance of each PC within the shape‐only model. Muscles are displayed in the orientation indicated by the reference shown in the bottom right corner of each illustration.

**FIGURE 4:**
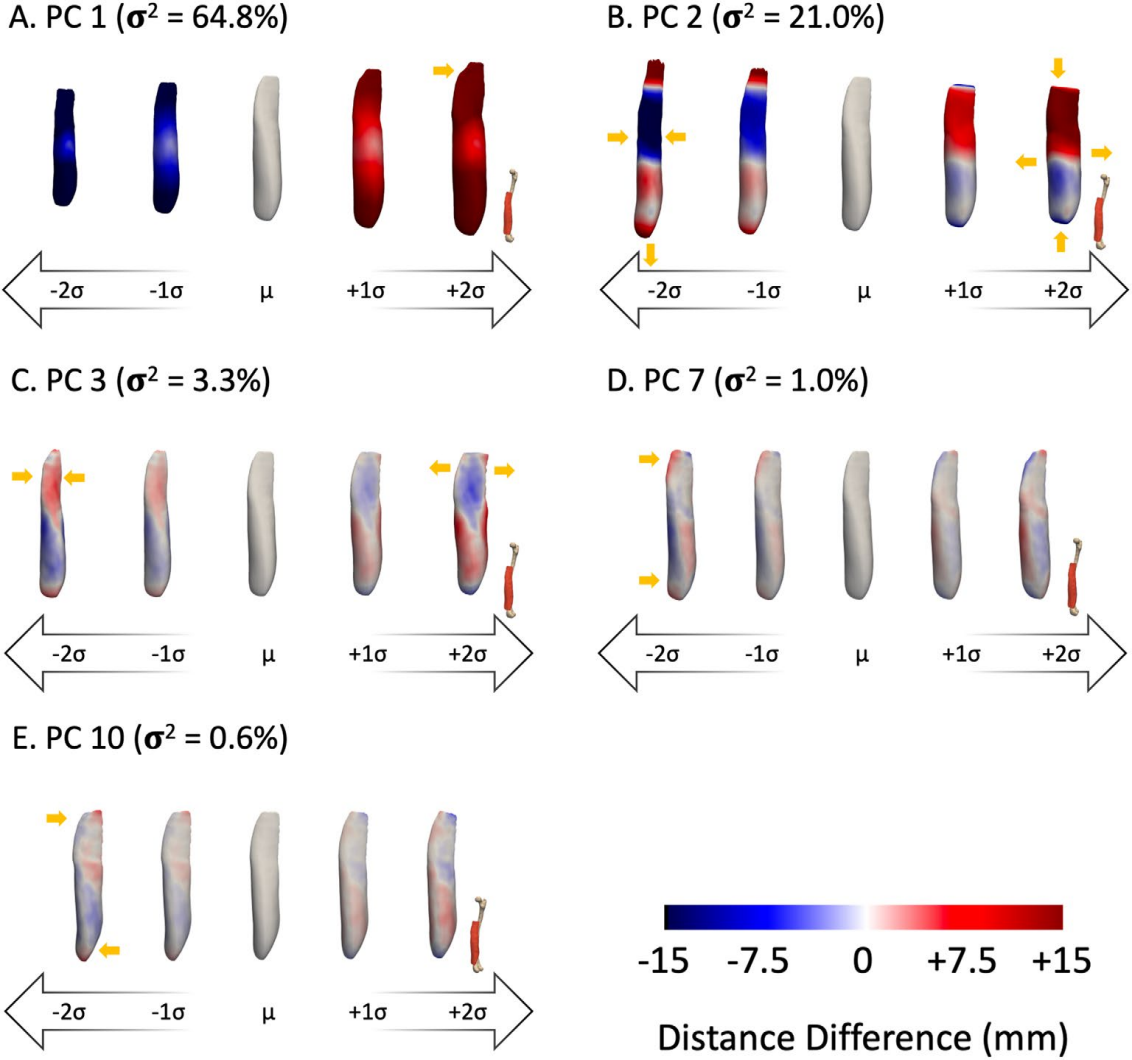
The figure shows five principal components (PCs 1, 2, 3, 7, and 10) of the VM muscle contributed to the prediction of concentric knee extension torque (significant shape‐only features, *p* < 0.05). The color‐coded maps represent surface‐to‐surface distance (in millimeters) between the muscle models at ±2 standard deviations and mean muscle. 𝛔^2^ represents the variance of each PC within the shape‐only model. Muscles are displayed in the orientation indicated by the reference shown in the bottom right corner of each illustration.

**FIGURE 5:**
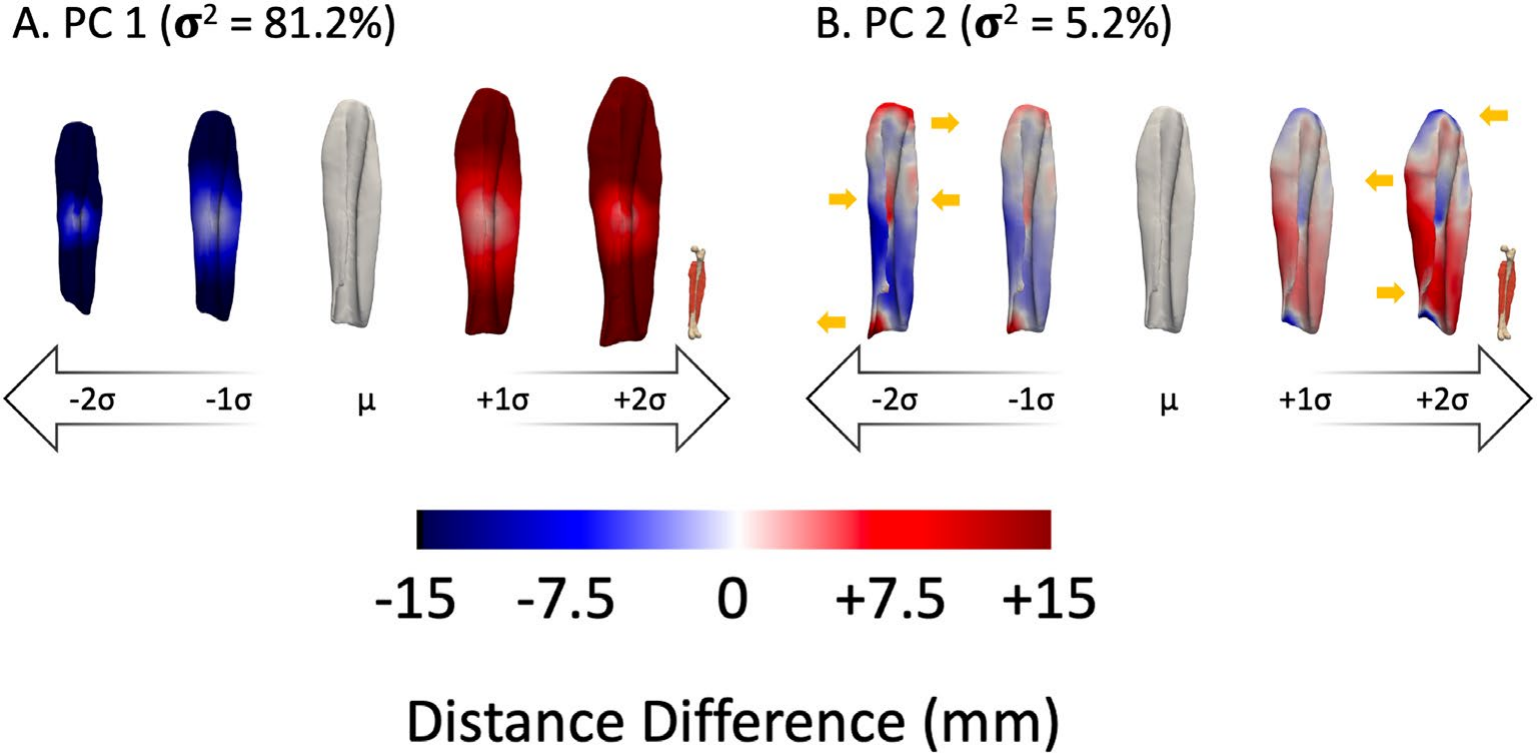
The figure shows two principal components (PCs 1 and 2) of the VI muscle contributed to the prediction of concentric knee extension torque (significant shape‐only features, *p* < 0.05). The color‐coded maps represent surface‐to‐surface distance (in millimeters) between the muscle models at ±2 standard deviations and mean muscle. 𝛔^2^ represents the variance of each PC within the shape‐only model. Muscles are displayed in the orientation indicated by the reference shown in the bottom right corner of each illustration.

Subsequent PCs of shape‐only models differ in their morphological representations, but the predominant shape across the four quadriceps muscles describes expansion versus compression at certain regions of the muscle, particularly the proximal region. These subsequent PCs accounted for independent variances of less than 4% for each PC of the shape‐only models—Figures 2, 3, 4, 5.

### 3.2 Shape + Architecture Models

Muscle architecture was combined with muscle shape information to generate shape + architecture models. This increased the cumulative variance of the first 10 PCs for the four investigated quadriceps muscles. The cumulative variance of the first 10 PCs was 96.6% for VL, 98.4% for RF, 97.8% for VM, and 96.4% for VI. The cumulative variances of shape + architecture models were larger than the cumulative variances of the corresponding shape‐only models.

**FIGURE 6:**
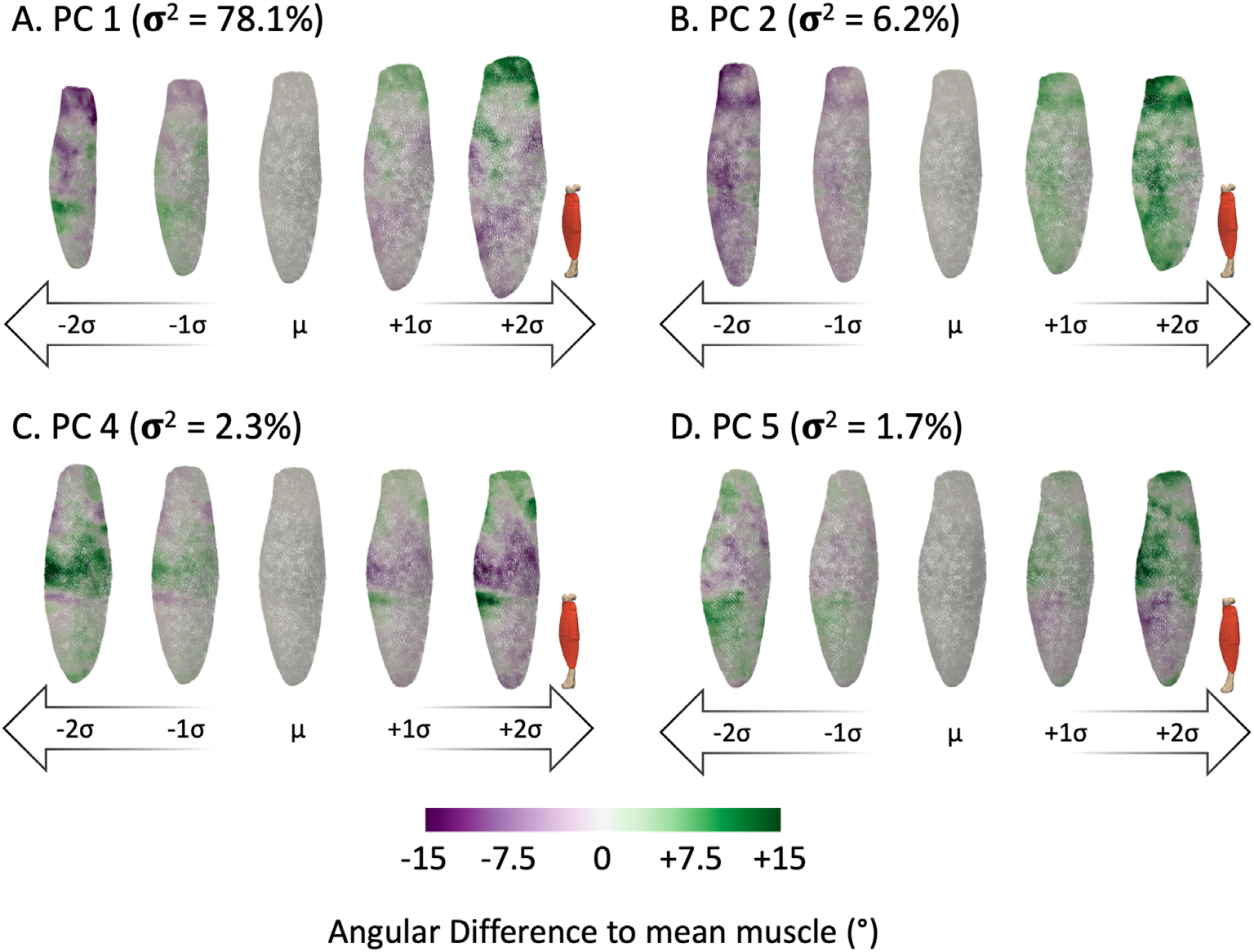
Four principal components (PCs 1, 2, 4, and 5) of the VL muscle contributed to the prediction of concentric knee extension torque (significant shape + architecture features, *p* < 0.05). The color‐coded maps represent vector‐to‐vector difference (in degrees) between the muscle models at ±2 standard deviations and mean muscle. 𝛔^2^ represents the variance of each PC within the shape + architecture model. Muscles in all panels are depicted in an anatomical anterior view, as shown in the orientation reference at the bottom right corner of each PC illustrations.

**FIGURE 7:**
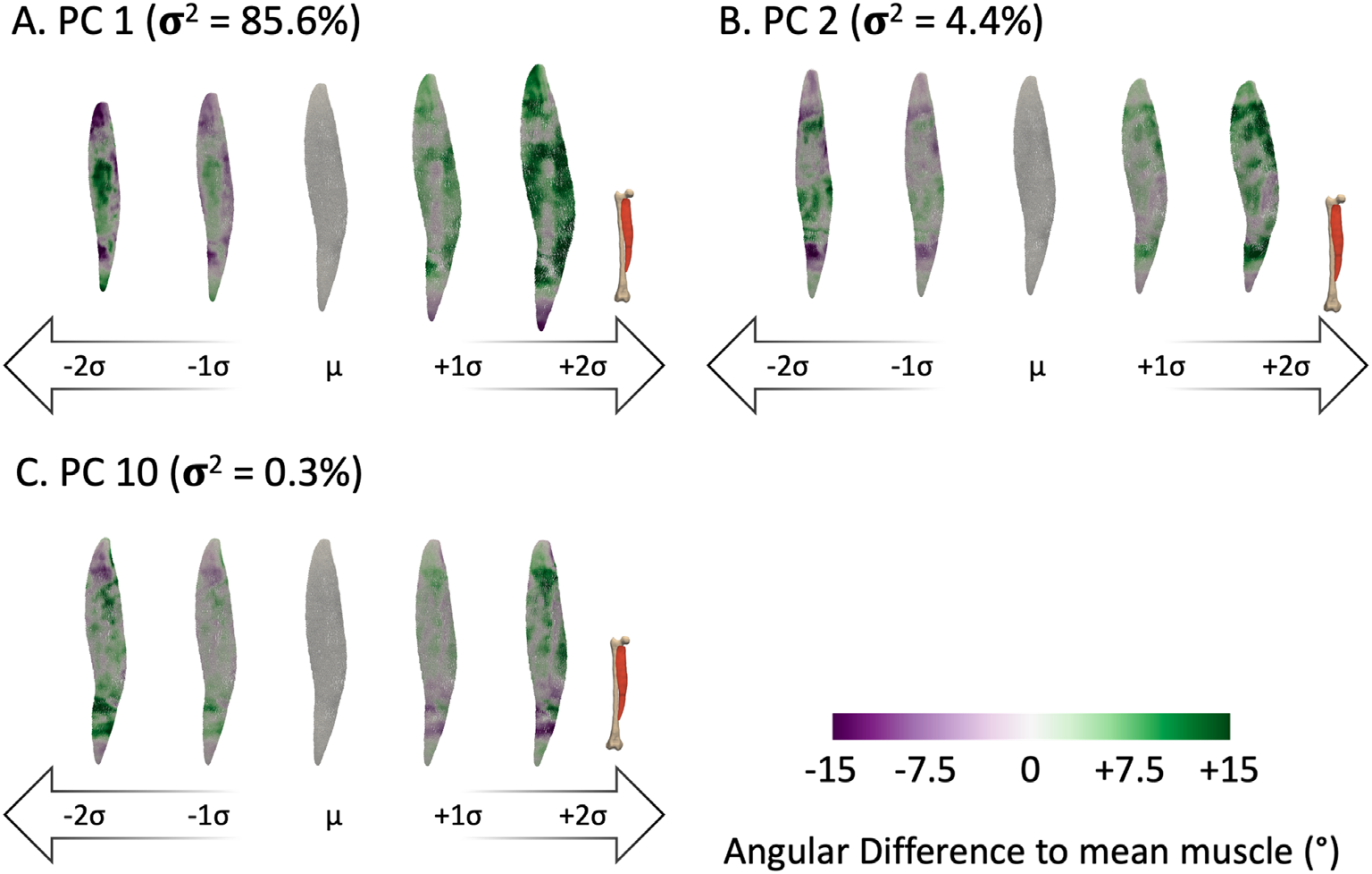
The figure shows three principal components (PCs 1, 2, and 10) of the RF muscle contributed to the prediction of concentric knee extension torque (significant shape + architecture features, *p* < 0.05). The color‐coded maps represent vector‐to‐vector difference (in degrees) between the muscle models at ±2 standard deviations and mean muscle. 𝛔^2^ represents the variance of each PC within the shape + architecture model. Muscles are displayed in the orientation indicated by the reference shown in the bottom right corner of each illustration.

**FIGURE 8:**
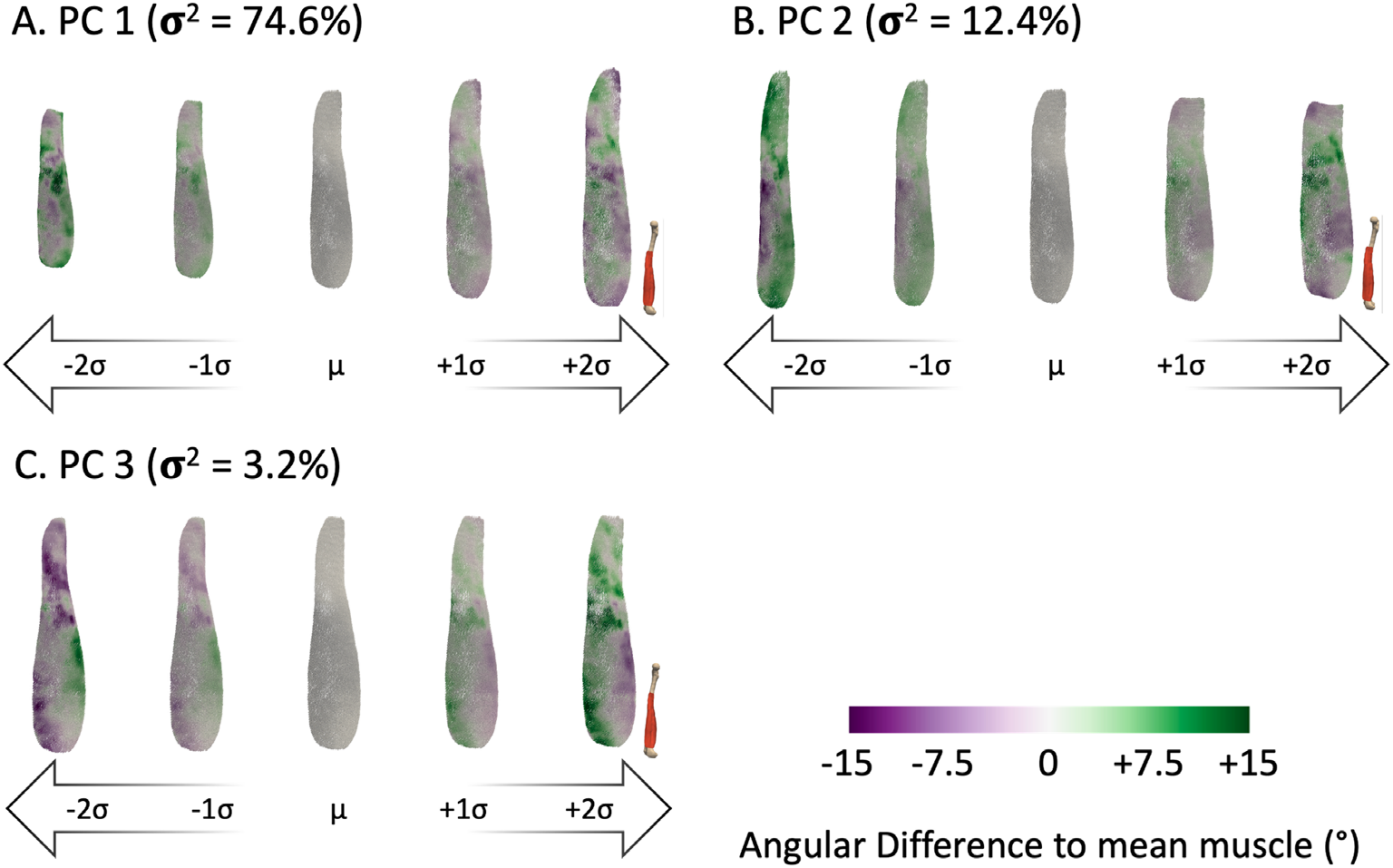
The figure shows four principal components (PCs 1, 2, and 3) of the VM muscle contributed to the prediction of concentric knee extension torque (significant shape + architecture features, *p* < 0.05). The color‐coded maps represent vector‐to‐vector difference (in degrees) between the muscle models at ±2 standard deviations and mean muscle. 𝛔^2^ represents the variance of each PC within the shape + architecture model. Muscles are displayed in the orientation indicated by the reference shown in the bottom right corner of each illustration.

**FIGURE 9:**
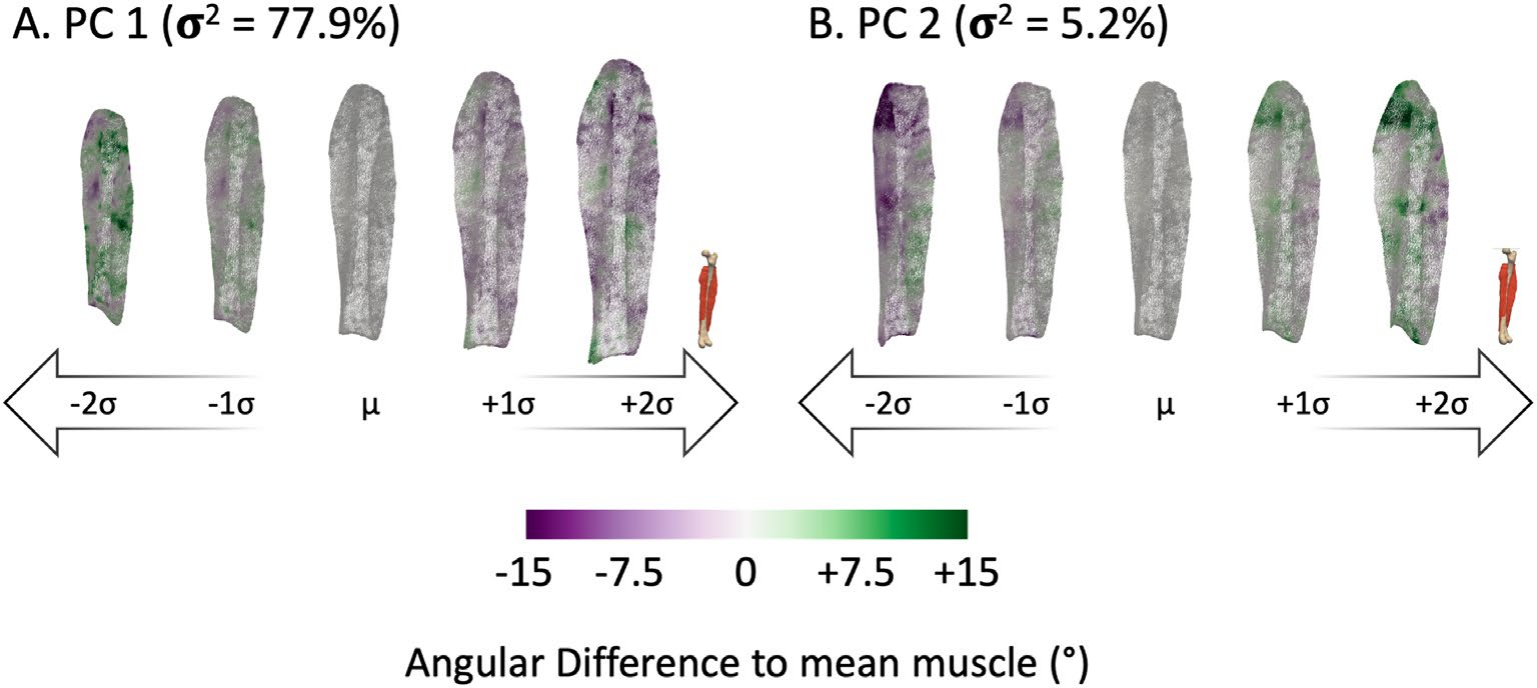
The figure shows two principal components (PCs 1 and 2) of the VI muscle contributed to the prediction of concentric knee extension torque (significant shape + architecture features, *p* < 0.05). The color‐coded maps represent vector‐to‐vector difference (in degrees) between the muscle models at ±2 standard deviations and mean muscle. 𝛔^2^ represents the variance of each PC within the shape + architecture model. Muscles are displayed in the orientation indicated by the reference shown in the bottom right corner of each illustration.

**FIGURE 10:**
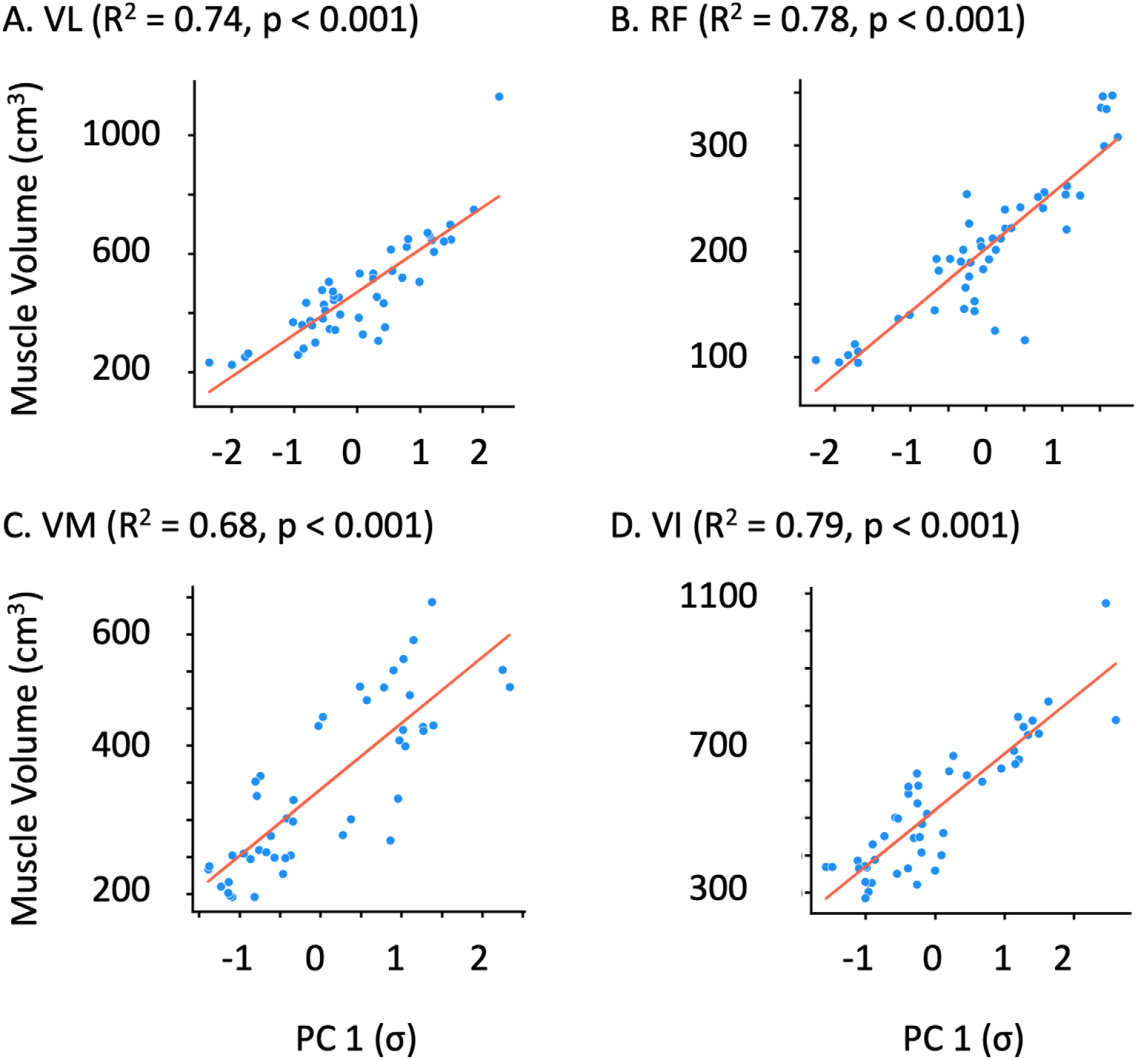
Correlation between the first principal component (PC 1) of the shape + architecture models and muscle volume. *R* ^2^ was 0.74 for VL, 0.78 for RF, 0.68 for VM, and 0.79 for VI. VL: Vastus lateralis, RF: Rectus femoris, VM: Vastus medialis, and VI: Vastus intermedius.

The PCs captured by shape + architecture models explained morphological features of the four investigated muscles—VL, RF, VM, and VI. These morphological features are the same as the features presented in the shape‐only models. Beyond the abovementioned morphological features and more importantly, the PCs of shape + architecture models demonstrated regional variations of local fiber orientations—Figures 6, 7, 8, 9. The variance of the first PC of the shape + architecture models was greater compared to their corresponding PC of the shape‐only models (78.1% for VL, 85.6% for RF, 74.6% for VM and 77.9% for VI). However, the correlation between the first PC of the shape + architecture models with muscle volume was weaker than for corresponding PCs of the shape‐only models (*R* ^2^ = 0.74 for VL, 0.78 for RF, 0.68 for VM, and 0.79 for VI)—Figure 10. The first PC vs. muscle volume correlation discrepancy may reveal no association between regional variations of fiber orientations and muscle volume. Furthermore, subsequent PCs of the shape + architecture models had a lower variance relative to their equivalent PCs in the shape‐only models.

For example, morphologically, the first PC of the shape + architecture model of the VL muscle described variations associated with muscle size. These morphological variations were, also, associated with regional variations of fiber orientations. At one end of this PC, fiber orientations—vector‐to‐vector difference—changed by more than 15^ο^ in the proximal region of the VL muscle, while at the other end of the same PC, fiber orientations changed by more than −15^ο^ in the proximal region of the VL muscle—Figure 6.

### 3.3 Torque Prediction

Prediction of knee extension torque using muscle volume was investigated in three different scenarios: (1) concentric, (2) isometric, and (3) eccentric. The *R* ^2^ values of the volume‐only models were considered as the baseline for the prediction performance. Outputs of in‐sample regression models were summarized in Table S2. The *R* ^2^ of the LOSO‐CV models—using muscle volume‐only as a predictor and multiple linear mixed‐effect regression model—were higher for the concentric and isometric torque compared to the eccentric torque for the VL, RF, and VM muscle. The prediction performance of the eccentric torque was weak to moderate for all four quadriceps muscles—Table 1.

**TABLE 1:** Coefficients of determinations (*R* ^2^) and root mean squared error (RMSE) with pairwise, subject‐level 95% confidence interval (CI) of the predicted and actual torque values obtained via the mixed‐effect models using (1) muscle volume, (2) shape‐only features, and (3) shape + architecture features.

		Muscle volume (cm^3^)		Shape‐only		Shape + architecture	
		*R* ^2^ (95% CI)	RMSE (Nm) (95% CI)	*R* ^2^ (95% CI)	RMSE (Nm) (95% CI)	*R* ^2^ (95% CI)	RMSE (Nm) (95% CI)
Concentric (120°/s)	VL	0.65 (0.31–0.82)	21.0 (15.9–25.7)	0.77 (0.56–0.87)	17.1 (13.4–20.4)	0.67 (0.36–0.83)	20.2 (14.9–24.9)
	RF	0.65 (0.41–0.77)	20.9 (16.2–25.4)	0.65 (0.44–0.76)	20.9 (16.0–25.3)	0.59 (0.23–0.76)	22.8 (17.0–27.9)
	VM	0.53 (0.03–0.76)	24.1 (17.1–31.6)	0.43 (−0.23–0.73)	26.6 (19.2–33.5)	0.38 (−0.31–0.72)	27.9 (18.7–36.4)
	VI	0.32 (−0.32–0.59)	29.1 (19.4–38.6)	0.47 (−0.16–0.74)	25.8 (19.0–34.1)	0.44 (−0.24–0.73)	26.4 (18.8–35.2)
Isometric (0°/s)	VL	0.71 (0.49–0.80)	26.6 (20.6–33.2)	0.71 (0.45–0.84)	26.4 (20.6–32.5)	0.69 (0.40–0.83)	27.4 (20.8–33.8)
	RF	0.60 (0.28–0.76)	30.9 (25.9–35.4)	0.58 (0.26–0.73)	31.8 (26.7–36.8)	0.53 (0.13–0.73)	33.6 (25.9–41.2)
	VM	0.64 (0.31–0.80)	29.3 (22.4–36.2)	0.60 (0.18–0.80)	31.0 (23.0–40.3)	0.63 (0.28–0.80)	29.8 (22.5–37.8)
	VI	0.43 (0.11–0.56)	37.1 (29.3–44.6)	0.61 (0.36–0.72)	30.6 (25.7–35.3)	0.57 (0.19–0.75)	32.4 (25.3–39.0)
Eccentric (−60°s)	VL	0.31 (0.02–0.45)	41.0 (32.8–49.2)	0.47 (0.16–0.64)	36.1 (28.2–44.0)	0.44 (0.09–0.63)	37.1 (29.0–45.1)
	RF	0.50 (0.18–0.67)	34.9 (27.2–42.5)	0.43 (0.04–0.65)	37.3 (27.8–46.7)	0.44 (0.03–0.68)	36.9 (27.1–46.8)
	VM	0.42 (0.04–0.64)	37.5 (28.5–45.8)	0.43 (−0.02–0.66)	37.3 (28.2–44.8)	0.40 (−0.01–0.62)	38.1 (29.5–45.5)
	VI	0.16 (−0.15–0.30)	45.1 (36.3–53.3)	0.29 (−0.04–0.45)	41.6 (33.6–49.8)	0.31 (−0.05–0.52)	40.9 (31.7–50.0)

*Note:* Shape‐only and shape + architecture features were excluded based on their significant contribution to the prediction models and were used to train mixed‐effect models using a leave‐one‐subject‐out (LOSO) cross validation (CV) approach. Models were used to predict (1) concentric, (2) isometric, and (3) eccentric knee extension torque for the four quadriceps muscles.

Abbreviations: RF: Rectus femoris; VI: Vastus intermedius; VL: Vastus lateralis; VM: Vastus medialis.

Pairwise, subject‐level bootstrapping comparisons revealed that the inclusion of PCs from shape‐only models statistically improved the model performance using shape‐only features of the VL muscle for eccentric torque—∆*R* ^2^ = 0.16 (0.01–0.29), ∆RMSE = 5.0 (0.4–9.7). Additionally, shape‐only features of the VI statistically improved the prediction of isometric torque—∆*R* ^2^ = 0.19 (0.02–0.36), ∆RMSE = 6.5 (0.7–12.0). Pairwise, subject‐level, bootstrapping results were summarized in Table 2. In contrast, shape + architecture models showed no statistically significant improvement in predicting concentric, isometric, or eccentric torque (all *p* ≥ 0.131).

**TABLE 2:** Pairwise, subject‐level comparison of *R* ^2^ and RMSE with 95% confidence interval (CI) of the cross‐validation models.

		Shape‐only versus volume		Shape + architecture versus volume	
		∆*R* ^2^ (95% CI)	∆RMSE (Nm) (95% CI)	∆*R* ^2^ (95% CI)	∆RMSE (Nm) (95% CI)
Concentric (120°/s)	VL	0.13 (0.00–0.32)	3.9 (0.0–7.9)	0.03 (−0.08–0.15)	0.8 (−2.2–3.9)
	RF	0.00 (−0.06–0.08)	0.1 (−1.8–2.0)	−0.07 (−0.28–0.08)	−1.8 (−6.0–2.3)
	VM	−0.11 (−0.50–0.18)	−2.4 (−8.9–4.4)	−0.17 (−0.53–0.03)	−3.6 (−9.3–0.8)
	VI	0.14 (−0.07–0.29)	3.2 (−1.4–8.1)	0.11 (−0.14–0.28)	2.7 (−2.3–7.7)
Isometric (0°/s)	VL	−0.00 (−0.09–0.07)	0.2 (−3.0–3.8)	−0.02 (−0.17–0.07)	−0.7 (−5.4–3.8)
	RF	−0.02 (−0.08–0.05)	−0.9 (−3.3–1.6)	−0.08 (−0.28–0.11)	−2.7 (−8.6–3.6)
	VM	−0.05 (−0.23–0.14)	−1.6 (−7.6–5.1)	−0.01 (−0.15–0.13)	−0.6 (−5.3–4.6)
	VI	0.19 (0.02–0.36)	6.5 (0.7–12.0)	0.13 (−0.13–0.33)	4.8 (−3.3–12.6)
Eccentric (−60°/s)	VL	0.16 (0.01–0.29)	5.0 (0.4–9.7)	0.13 (−0.10–0.32)	4.0 (−2.5–10.2)
	RF	−0.08 (−0.23–0.06)	−2.4 (−6.7–2.0)	−0.06 (−0.25–0.10)	−1.9 (−7.3–3.6)
	VM	0.00 (−0.22–0.22)	0.3 (−5.7–6.6)	−0.02 (−0.24–0.21)	−0.5 (−6.7–6.2)
	VI	0.13 (−0.04–0.30)	3.5 (−0.9–8.1)	0.15 (−0.07–0.36)	4.3 (−1.8–10.1)

*Note:* The models included comparison between the use of muscle volume, shape‐only, and shape + architecture the for the prediction of (1) concentric, (2) isometric, and (3) eccentric knee extension torque for the four quadriceps muscle. Positive *R* ^2^ and RMSE indicate an improvement in the model performance. 95% CI that does not cross zero indicates statistically significant improvement of the model.

Abbreviations: RF: Rectus femoris; VI: Vastus intermedius; VL: Vastus lateralis; VM: Vastus medialis.

The shape features beyond size had weak correlations with muscle volume—Figures S1 and S2. The removal of size‐related shape feature from the models—in which models were adjusted for volume—showed an increased *R* ^2^ for VL and VI models using shape‐only features compared to volume—Table S3. However, the effect size analysis did not show statistical significance (all *p* ≥ 0.073)—Table S4. Additionally, the inclusion of age, sex, and BMI in both shape‐only and shape + architecture models showed that they were not significant covariates (all *p* ≥ 0.089), with the exception of age being a significant covariate for the shape‐only and shape + architecture models of the VI muscle only. Bland–Altman plots revealed reduced proportional bias and lower limits of agreement for shape‐only models compared to volume‐only models, for both VL and VI muscles—Figures S3 and S4.

To explore the reason behind the lower *R* ^2^ and higher RMSE for the eccentric torque models compared to the concentric torque, residual diagnostics was conducted. The analysis of residual diagnostics revealed that for the VL muscle, the model of concentric torque using shape‐only features was more stable compared to the model of the eccentric torque using shape‐only. Unlike eccentric torque, concentric torque demonstrated that the distribution of residuals was approximately centered around zero and the normality assumption was reasonably satisfied with a few outlines suggesting acceptable model fit—Figures S5 and S6.

## 4 Discussion

In this study, we demonstrated that shape‐only features from quadriceps shape‐modeling, obtained from MR images, improved the prediction of muscle strength compared to models based on (1) muscle volume, and (2) shape + architecture. Both shape‐only models and shape + architecture models captured a large proportion of the total variance of each of the quadriceps, with a high cumulative variance of the first 10 PCs reflecting a good compactness of the developed models—that is, a few independent features or PCs can explain large variance of the muscle shape variability.

The first PC in both models primarily represented muscle size‐associated variations and accounted for the largest proportion of variance, indicative of the diverse range of muscle sizes within this study population. Despite differences in study populations and targeted muscles, these results are in alignment with previous results from shape‐only models of skeletal muscles, where the first PC represented muscle size variations [18, 19, 20]. For instance, Umehara et al. developed shape models of the quadriceps and demonstrated a size‐related first PC similar to our study regardless of muscle scaling. Additional PCs observed in this study (including curvature of the rectus femoris and narrowing and widening of the proximal region of the vastus intermedius muscle) were consistent with a previous study [20]. It is important to note that no volume scaling was applied to the muscles in this study for two reasons: (1) muscle size is important for muscle force production and (2) muscle volume was not included as a predictor in combination with shape‐only or shape + architecture features.

The shape‐only features of VL and VI muscle significantly improved the prediction performance of torque. While the first PC correlated with muscle volume, other PCs beyond size (PC2 and above) showed weak correlations with volume indicating their non‐size contribution to the model. The assessment of shape features beyond size (PC2 and above corrected for muscle volume) indicated non‐statistically significant improvement compared to muscle volume models for the VL muscle (all *p* ≥ 0.073). This may indicate that there is insufficient evidence that non‐size related features of the VL muscle provide substantial unique information beyond volume. This is illustrated by the weak correlations between muscle volume and shape features beyond size.

Additionally, the Bland–Altman analysis showed a reduced proportional bias in the prediction of concentric torque using shape‐only features compared to muscle volume. However, the overestimation and underestimation of the torque values at the two ends of the range requires further investigation to explore the reasons behind this phenomenon. The analysis of residual diagnostics revealed that for the VL muscle, the model of concentric torque using shape‐only features was more stable compared to the corresponding model for eccentric torque, as indicated by the fact that the distribution of residuals was approximately centered around zero and the normality assumption was reasonably satisfied—Figures S5 and S6. On the other hand, all models (volume, shape‐only, and shape + architecture features) explained less variance of the eccentric torque compared to both concentric and isometric torque. This higher variability is potentially due to the difficulty associated with measuring eccentric torque which requires a higher demand for skill and motor control of the subjects [35].

The inclusion of muscle architectural information to shape‐only features improved the compactness of the overall shape + architecture model but did not significantly improve predictions of knee extension torque beyond muscle volume (all *p* ≥ 0.131). The variations of fiber orientations were localized at certain regions of the muscles and are associated with variations in muscle shape, but the functional interpretation of these regional variations still remains unclear. Interestingly, the first PC derived from the shape + architecture model was not strongly associated with muscle volume. This suggests that the variance of muscle fiber orientation within the study cohort does not explain differences in muscle strength, warranting further investigation into their independent functional implications. The superior performance of the shape‐only model over shape + architecture models is likely because architectural variations were indirectly captured by shape‐only features. This indicates that shape‐only features, which can be easily obtained from any anatomical MRI or CT scan, offer a powerful and accessible approach for the assessment of muscle strength and allow for early detection of muscle weakness and sarcopenia.

The aim of this study was to identify imaging‐based biomarkers of muscle shape and architecture to improve muscle strength prediction compared to muscle volume. Blazevich et al. reported that muscle volume was the strongest predictor of isometric extensor moment compared to other muscle parameters like anatomical cross‐sectional area, physiological cross‐sectional area, moment arm, fascicle length, and fascicle angle [36]. Likewise, the multiple linear regression models in this study showed that the first PC, which correlates with muscle volume, was the strongest predictor of muscle strength compared to other subsequent PCs per each model. However, other muscle morphological features of the VL, RF, VM, and VI enhanced the prediction performance of our models compared to models using muscle volume as the only predictor. This is likely due to the rearrangement of muscle fibers resulting from remodeling of muscle architecture and affecting muscle shape, which underscores the value of muscle shape features in predicting muscle strength.

The findings of this study have broader implications for studying strength in older populations, individuals with muscle diseases, and identifying subjects at risk of sarcopenia. Importantly, our results suggest that muscle strength information can be derived from routine medical imaging, suggesting a potential “opportunistic screening” method for sarcopenia in patients undergoing MRI for unrelated conditions, such as rheumatic disease and trauma. While the segmentations in our study were performed manually, recent developments in deep learning enabled automatic muscle segmentation [26, 37, 38], and therefore offer the opportunity to streamline the extraction of shape information from imaging data. The combination of automatic segmentation and shape modeling will allow for automatic assessment of muscle shape and torque prediction. Implementing these combined approaches will enhance clinical reliability and utility, and will allow for early detection of sarcopenia. Early detection of sarcopenia can support timely interventions to mitigate morbidity and improve patient outcomes.

### 4.1 Limitations

One of the limitations of this study is that we included only healthy subjects and the results may not be generalized to people with muscle conditions like sarcopenia. Nonetheless, establishing imaging‐based features of healthy skeletal muscles can serve as a reference for detecting pathological deviations. A limitation of this study is the modest sample size and the use of bilateral measurements from the same individuals. Despite accounting for this using mixed‐effect models, the relatively small sample size may limit generalizability of this work. DTI acquisition relying on 15 diffusion directions provided acceptable signal‐to‐noise ratios (SNR) for estimating quadriceps architecture, but higher angular resolution diffusion MRI might yield more precise architectural metrics and improve strength prediction. An additional limitation is that we combined coordinates units (mesh vertices) and unit‐vector units (fiber orientations) despite their different scales. However, this approach led to more compact prediction models while preserving the structural and physical interpretability of the components, as observed in Zhang et al. [23]. Another limitation relates to the intrinsic deformability of soft tissues such as skeletal muscle due to motion and external pressure. This concern was mitigated by relatively fast acquisition sequences, and consistent subject positioning. Lastly, access to larger and diverse datasets combined with an automated pipeline would further improve the performance of muscle strength prediction models and enhance the generalizability and reproducibility of these findings.

### 4.2 Conclusions

This study confirms the importance of muscle shape features in force generation properties of skeletal muscles. The findings of this study support the hypothesis that combining shape modeling with imaging data improves muscle strength prediction compared to volume‐only approaches, paving the way for the development of imaging‐based biomarkers for muscular health. Routine MRI or CT scans could be leveraged for “opportunistic screening” to assess muscle shape and function, enabling early detection of sarcopenia or other muscular diseases. Prior to clinical translation, models will require external validation in multi‐center datasets, and development of shape‐only and shape + architecture models of populations with muscular conditions like sarcopenia. Future research should explore the integration of muscle shape features with muscle biomechanics to study architectural remodeling in older adults, which may help identify individuals at risk for sarcopenia.

## Funding

This study was supported by NIH Grants R21AG093093, R00AG071735 and R01EB002524.

## Disclosure

The authors have nothing to report.

## Conflicts of Interest

The authors declare no conflicts of interest.

## Supporting information


**Table S1:** Mean ± SD of dice score (%), IoU (%), and ASSD (mm) metrics of the inter‐rater variability (manual segmentation vs semi‐automatic segmentation). The dice score values (%) ranges between 81.7 ± 2.7 for the vastus intermedius and 88.4 ± 4.8 for the rectus femoris which indicates acceptable inter‐rater variability.
**Table S2:** The table below presents the outputs of in‐sample regression models. Squared Pearson correlation (*R* ^2^) and root mean squared error (RMSE) of the predicted vs actual torque values obtained via the mixed‐effect models using (1) muscle volume, (2) shape‐only features, and (3) shape + architecture features. Selected features which are statistically significant via shape‐only and shape + architecture models were used in the mixed‐effect models. Models were used to predict (1) concentric, (2) isometric, and (3) eccentric knee extension torque. VL: vastus lateralis, RF: rectus femoris, VM: vastus medialis, VI: vastus intermedius.
**Table S3:** The table below shows the outputs of the mixed‐effect models using (1) muscle volume, (2) shape‐only features beyond size‐related feature (PC2 and above) adjusted for muscle volume, and (3) shape + architecture features beyond size‐related feature (PC2 and above) adjusted for muscle volume. Coefficients of determinations (*R* ^2^) and root mean squared error (RMSE) with 95% confidence interval (CI) of the predicted vs actual torque values obtained via the mixed‐effect models. Selected features which are statistically significant via shape‐only and shape + architecture models were used to train the mixed‐effect models. Models were used to predict (1) concentric, (2) isometric, and (3) eccentric knee extension torque and evaluated using leave‐one‐subject‐out (LOSO), cross validation (CV). VL: vastus lateralis, RF: rectus femoris, VM: vastus medialis, VI: vastus intermedius. N/A indicates that here is no shape features beyond size contributed significantly to the model.
**Table S4:** Pairwise, subject‐level comparison of *R* ^2^ and RMSE with 95% confidence interval (CI) of the cross‐validation models using shape features beyond size and adjusted for volume. The models included comparison between the use of muscle volume, shape‐only, and shape + architecturethe for the prediction of (1) concentric, (2) isometric, and (3) eccentric knee extension torque for the four quadriceps muscle. Positive *R* ^2^ and RMSE indicate an improvement in the model performance. 95% CI that does not cross zero indicates statistically significant improvement of the model. VL: vastus lateralis, RF: rectus femoris, VM: vastus medialis, and VI: vastus intermedius.
**Figure S1:** The Pearson correlation coefficient (r) of subjects' characteristics and shape features—PC1, PC2, and PC4—contributing to the prediction model of concentric torque of the vastus lateralis (VL) muscle. Only correlations with *p* < 0.05 are shown.
**Figure S2:** The Pearson correlation coefficient (r) of subjects’ characteristics and shape features—PC1, PC2 and PC9—contributing to the prediction model of isometric torque of the vastus intermedius (VI) muscle. Only correlations with *p* < 0.05 are shown.
**Figure S3:** The Bland‐Altman Plots of the predicted and actual concentric knee extension torque using (a) muscle volume model and (b) shape‐only model of the vastus lateralis (VL) muscle.
**Figure S4:** The Bland‐Altman Plots of the predicted and actual isometric knee extension torque using (a) muscle volume model, and (b) shape‐only model of the vastus intermedius (VI) muscle.
**Figure S5:** Residual diagnostics of shape‐only features of the vastus lateralis (VL) predicting eccentric torque. The figure includes the residuals‐versus‐fitted plot, histogram of residuals and Q‐Q plot to evaluate the assumptions of linearity, homoscedasticity, normality of residuals and influential outliers.
**Figure S6:** Residual diagnostics of shape‐only features of the vastus lateralis (VL) predicting concentric torque. The figure includes the residuals‐versus‐fitted plot, histogram of residuals and Q‐Q plot to evaluate the assumptions of linearity, homoscedasticity, normality of residuals and influential outliers.
