# Supplementary material for: MR Imaging‐Based Biomarkers for Strength Prediction: A Statistical Shape and Architecture Modeling of Quadriceps Muscles
Source: J Magn Reson Imaging. 2026 Jun 11;64(3):685–98. doi: 10.1002/jmri.70377 (PMC13466587; doi:10.1002/jmri.70377)
Supplement: Supplementary file 1 [file JMRI-64-685-s001.docx]

**Supplementary Materials**

**TABLE 1 | Mean ± SD of dice score (%), IoU (%), and ASSD (mm) metrics of the inter-rater variability (manual segmentation vs semi-automatic segmentation). The dice score values (%) ranges between 81.7 ± 2.7 for the vastus intermedius and 88.4 ± 4.8 for the rectus femoris which indicates acceptable inter-rater variability.**

|  | **Dice (Mean ± SD)**  **(%)** | **IoU (Mean ± SD)**  **(%)** | **ASSD (Mean ± SD)**  **(mm)** |
| --- | --- | --- | --- |
| **Vastus Lateralis (VL)** | 85.6 ± 3.6 | 75.0 ± 5.5 | 1.6 ± 0.52 |
| **Rectus Femoris (RF)** | 88.4 ± 4.8 | 79.5 ± 7.0 | 1.3 ± 1.1 |
| **Vastus Medialis (VM)** | 87.8 ± 2.3 | 78.4 ± 3.6 | 1.8 ± 0.93 |
| **Vastus Intermedius (VI)** | 81.7 ± 2.7 | 69.1 ± 3.9 | 2.3 ± 0.63 |

**TABLE 2 |** The table below presents the outputs of *in-sample regression models*. Squared Pearson correlation (R^2^) and root mean squared error (RMSE) of the predicted vs actual torque values obtained via the mixed-effect models using 1) muscle volume, 2) shape-only features, and 3) shape+architecture features. Selected features which are statistically significant via shape-only and shape+architecture models were used in the mixed-effect models. Models were used to predict 1) concentric, 2) isometric, and 3) eccentric knee extension torque. VL: vastus lateralis, RF: rectus femoris, VM: vastus medialis, VI: vastus intermedius.

|  | | **Muscle volume (cm3)** | | | **Shape-only** | | | **Shape+architecture** | |
| --- | --- | --- | --- | --- | --- | --- | --- | --- | --- |
|  |  | **R^2^** | **RMSE (Nm)** | **R^2^** | | **RMSE (Nm)** | **R^2^** | | **RMSE (Nm)** |
| **Concentric**  **(120 o /s)** | **VL** | 0.68 | 20.0 | 0.81 | | 15.6 | 0.77 | | 16.9 |
|  | **RF** | 0.69 | 19.7 | 0.70 | | 19.4 | 0.67 | | 20.3 |
|  | **VM** | 0.59 | 22.7 | 0.62 | | 22.2 | 0.55 | | 23.6 |
|  | **VI** | 0.45 | 26.6 | 0.57 | | 23.3 | 0.55 | | 23.8 |
| **Isometric**  **(0 o /s)** | **VL** | 0.76 | 24.5 | 0.76 | | 23.8 | 0.76 | | 24.3 |
|  | **RF** | 0.65 | 29.0 | 0.64 | | 29.3 | 0.61 | | 30.7 |
|  | **VM** | 0.69 | 27.3 | 0.71 | | 26.4 | 0.75 | | 24.5 |
|  | **VI** | 0.53 | 34.4 | 0.70 | | 27.1 | 0.68 | | 27.8 |
| **Eccentric**  **(-60 o /s)** | **VL** | 0.46 | 37.5 | 0.56 | | 32.8 | 0.53 | | 33.8 |
|  | **RF** | 0.56 | 32.9 | 0.49 | | 35.3 | 0.50 | | 34.8 |
|  | **VM** | 0.49 | 35.3 | 0.59 | | 31.7 | 0.54 | | 33.4 |
|  | **VI** | 0.31 | 41.9 | 0.42 | | 37.8 | 0.43 | | 37.7 |

**TABLE 3 |** The table below shows the outputs of the mixed-effect models using 1) muscle volume, 2) shape-only features beyond size-related feature (PC2 and above) adjusted for muscle volume, and 3) shape+architecture features beyond size-related feature (PC2 and above) adjusted for muscle volume. Coefficients of determinations (R^2^) and root mean squared error (RMSE) with 95% confidence interval (CI) of the predicted vs actual torque values obtained via the mixed-effect models. Selected features which are statistically significant via shape-only and shape+architecture models were used to train the mixed-effect models. Models were used to predict 1) concentric, 2) isometric, and 3) eccentric knee extension torque and evaluated using leave-one-subject-out (LOSO), cross validation (CV). VL: vastus lateralis, RF: rectus femoris, VM: vastus medialis, VI: vastus intermedius. N/A indicates that here is no shape features beyond size contributed significantly to the model.

|  | | **Muscle volume (cm3)** | | | **shape-only**  **(non-size features)** | | | **shape+architecture**  **(non-size features)** | |
| --- | --- | --- | --- | --- | --- | --- | --- | --- | --- |
|  |  | **R^2^**  **(95% CI)** | **RMSE (Nm)**  **(95% CI)** | **R^2^**  **(95% CI)** | | **RMSE (Nm)**  **(95% CI)** | **R^2^**  **(95% CI)** | | **RMSE (Nm)**  **(95% CI)** |
| **Concentric**  **(120 o /s)** | **VL** | 0.65  (0.31–0.82) | 21.0  (15.9–25.7) | 0.72  (0.52–0.81) | | 18.8  (15.5–21.7) | 0.65  (0.39–0.78) | | 21.0  (16.9–24.9) |
|  | **RF** | 0.65  (0.41–0.77) | 20.9  (16.2–25.4) | 0.64  (0.39–0.77) | | 21.1  (16.1–25.9) | 0.60  (0.33–0.75) | | 22.3  (17.7–26.5) |
|  | **VM** | 0.53  (0.03–0.76) | 24.1  (17.1–31.6) | 0.52  (-0.00–0.76) | | 24.5  (18.0–30.5) | 0.48  (-0.13–0.77) | | 25.6  (17.0–33.6) |
|  | **VI** | 0.32  (-0.32–0.59) | 29.1  (19.4–38.6) | 0.37  (-0.19–0.59) | | 28.1  (21.1–35.6) | 0.41  (-0.15–0.65) | | 27.1  (19.6–35.6) |
| **Isometric**  **(0 o /s)** | **VL** | 0.71  (0.49–0.80) | 26.6  (20.6–33.2) | 0.66  (0.46–0.76) | | 28.4  (22.4–35.0) | 0.66  (0.44–0.76) | | 28.8  (22.1–35.9) |
|  | **RF** | 0.60  (0.28–0.76) | 30.9  (25.9–35.4) | 0.59  (0.26–0.76) | | 31.3  (26.1–36.0) | 0.59  (0.23–0.76) | | 31.5  (26.7–36.2) |
|  | **VM** | 0.64  (0.31–0.80) | 29.3  (22.4–36.2) | 0.65  (0.27–0.82) | | 29.2  (21.4–38.2) | 0.66  (0.33–0.83) | | 28.4  (21.2–36.0) |
|  | **VI** | 0.43  (0.11–0.56) | 37.1  (29.3–44.6) | 0.58  (0.36–0.68) | | 31.9  (24.9–38.9) | 0.50  (0.15–0.68) | | 34.5  (26.3–42.8) |
| **Eccentric**  **(-60 o /s)** | **VL** | 0.31  (0.02–0.45) | 41.0  (32.8–49.2) | 0.30  (0.01–0.47) | | 41.2  (32.3–50.2) | 0.32  (-0.03–0.48) | | 40.9  (31.6–50.6) |
|  | **RF** | 0.50  (0.18–0.67) | 34.9  (27.2–42.5) | 0.50  (0.18–0.67) | | 34.9  (27.2–42.5) | 0.54  (0.23–0.70) | | 33.6  (26.4–41.1) |
|  | **VM** | 0.42  (0.04–0.64) | 37.5  (28.5–45.8) | 0.46  (0.06–0.69) | | 36.2  (26.7–44.4) | 0.45  (0.06–0.67) | | 36.6  (27.9–44.1) |
|  | **VI** | 0.16  (-0.15–0.30) | 45.1  (36.3–53.3) | 0.26  (-0.08–0.43) | | 42.4  (34.9–50.6) | 0.31  (-0.01–0.47) | | 41.0  (33.4–48.8) |

**TABLE 4 |** Pairwise, subject-level comparison of R^2^ and RMSE with 95% confidence interval (CI) of the cross-validation models using shape features beyond size adjusted for volume. The models included comparison between the use of muscle volume, shape-only, and shape+architecturethe for the prediction of 1) concentric, 2) isometric, and 3) eccentric knee extension torque for the four quadriceps muscle. Positive R^2^ and RMSE indicate an improvement in the model performance. 95% CI that does not cross zero indicates statistically significant improvement of the model. VL: vastus lateralis, RF: rectus femoris, VM: vastus medialis, and VI: vastus intermedius.

|  | | **Shape-only vs. Volume** | | **Shape+architecture vs. Volume** | |
| --- | --- | --- | --- | --- | --- |
|  |  | ∆**R^2^**  **(95% CI)** | ∆**RMSE (Nm)**  **(95% CI)** | ∆**R^2^**  **(95% CI)** | ∆**RMSE (Nm)**  **(95% CI)** |
| **Concentric**  **(120** o **/s)** | **VL** | 0.08  (-0.07–0.28) | 2.1  (-2.4–6.4) | 0.00  (-0.10–0.14) | -0.1  (-3.4–3.2) |
|  | **RF** | -0.01  (-0.03–0.01) | -0.2  (-0.8–0.2) | -0.05  (-0.15–0.03) | -1.3  (-3.7–0.8) |
|  | **VM** | -0.02  (-0.28–0.21) | -0.4  (-5.4–4.7) | -0.07  (-0.29–0.07) | -1.4  (-5.6–1.8) |
|  | **VI** | 0.04  (-0.19–0.23) | 0.9  (-3.7–4.7) | 0.09  (-0.09–0.27) | 2.0  (-1.8–5.7) |
| **Isometric**  **(0** o **/s)** | **VL** | -0.04  (-0.07–-0.02) | -1.8  (-3.2–-0.6) | -0.05  (-0.08–-0.03) | -2.2  (-3.6–-1.0) |
|  | **RF** | -0.01  (-0.02–-0.00) | -0.4  (-0.7–-0.1) | -0.02  (-0.10–0.04) | -0.7  (-3.1–1.3) |
|  | **VM** | 0.00  (-0.14–0.13) | 0.1  (-4.8–4.8) | 0.02  (-0.09–0.15) | 0.8  (-3.1–5.2) |
|  | **VI** | 0.16  (-0.01–0.39) | 5.3  (-0.4–11.3) | 0.08  (-0.20–0.31) | 2.6  (-5.4–9.9) |
| **Eccentric**  **(-60** o **/s)** | **VL** | -0.01  (-0.16–0.13) | -0.2  (-4.1–3.5) | 0.01  (-0.24–0.21) | 0.2  (-6.3–5.6) |
|  | **RF** | N/A | N/A | 0.04  (-0.02–0.09) | 1.2  (-0.6–2.9) |
|  | **VM** | 0.04  (-0.13–0.21) | 1.4  (-3.6–6.6) | 0.03  (-0.14–0.21) | 1.0  (-4.1–6.1) |
|  | **VI** | 0.10  (-0.11–0.31) | 2.7  (-2.6–8.2) | 0.15  (-0.01–0.33) | 4.1  (-0.3–9.2) |

**FIGURE 1** | The Pearson correlation coefficient (r) of subjects’ characteristics and shape features —PC1, PC2, and PC4— contributing to the prediction model concentric torque of the vastus lateralis (VL) muscle. Only correlations with p < 0.05 are shown.

**FIGURE 2** | The Pearson correlation coefficient (r) of subjects’ characteristics and shape features —PC1, PC2 and PC9—contributing to the prediction model of isometric torque of the vastus intermedius (VI) muscle. Only correlations with p < 0.05 are shown.

**FIGURE 3 |** The Bland-Altman Plots of the predicted and actual concentric knee extension torque using a) muscle volume model and b) shape-only model of the vastus lateralis (VL) muscle.

**FIGURE 4 |** The Bland-Altman Plots of the predicted and actual isometric knee extension torque using a) muscle volume model, and b) shape-only model of the vastus intermedius (VI) muscle.

**Figure 5** | Residual diagnostics of shape-only features of the vastus lateralis (VL) predicting eccentric torque. The figure includes the residuals-versus-fitted plot, histogram of residuals and Q-Q plot to evaluate the assumptions of linearity, homoscedasticity, normality of residuals and influential outliers.

**Figure 6 |** Residual diagnostics of shape-only features of the vastus lateralis (VL) predicting concentric torque. The figure includes the residuals-versus-fitted plot, histogram of residuals and Q-Q plot to evaluate the assumptions of linearity, homoscedasticity, normality of residuals and influential outliers.
